# Application of Species Distribution Modeling for Avian Influenza surveillance in the United States considering the North America Migratory Flyways

**DOI:** 10.1038/srep33161

**Published:** 2016-09-14

**Authors:** Jaber Belkhiria, Moh A. Alkhamis, Beatriz Martínez-López

**Affiliations:** 1 Center for Animal Disease Modeling and Surveillance, Department of Medicine & Epidemiology, School of Veterinary Medicine, University of California Davis, California, United States of America; 2 Environmental & Life Sciences Research Center, Kuwait Institute for Scientific Research, Kuwait; 3 Veterinary Population Medicine Department, Veterinary Medical Center, University of Minnesota, St. Paul, Minnesota, United States of America

## Abstract

Highly Pathogenic Avian Influenza (HPAI) has recently (2014–2015) re-emerged in the United States (US) causing the largest outbreak in US history with 232 outbreaks and an estimated economic impact of $950 million. This study proposes to use suitability maps for Low Pathogenic Avian Influenza (LPAI) to identify areas at high risk for HPAI outbreaks. LPAI suitability maps were based on wild bird demographics, LPAI surveillance, and poultry density in combination with environmental, climatic, and socio-economic risk factors. Species distribution modeling was used to produce high-resolution (cell size: 500m x 500m) maps for Avian Influenza (AI) suitability in each of the four North American migratory flyways (NAMF). Results reveal that AI suitability is heterogeneously distributed throughout the US with higher suitability in specific zones of the Midwest and coastal areas. The resultant suitability maps adequately predicted most of the HPAI outbreak areas during the 2014–2015 epidemic in the US (i.e. 89% of HPAI outbreaks were located in areas identified as highly suitable for LPAI). Results are potentially useful for poultry producers and stakeholders in designing risk-based surveillance, outreach and intervention strategies to better prevent and control future HPAI outbreaks in the US.

Avian Influenza (AI) is an infectious viral disease of domestic and wild birds. Depending on the strain and severity of the clinical symptoms on naïve chickens, AI is pathotyped into low pathogenic avian influenza (LPAI) or highly pathogenic avian influenza (HPAI)^1^. Mild symptoms are observed in poultry infected with LPAI and wild birds usually are asymptomatic. HPAI however, is a re-emerging, highly contagious and economically devastating viral infection with severe socio-economic consequences that strongly impact the poultry industry^2^. HPAI is also considered an important public health concern due to the potential contribution of AI virus (AIV) to the emergence of human influenza pandemics^3^. So far, subtypes H5 and H7 of the virus are recognized to cause HPAI, but not all H5 and H7 serotypes are virulent^4^. It has been proven that AI viruses (AIV) undergo frequent genetic re-assortment and LPAI may mutate to HPAI^5^. Little is known about the origin of LPAI to HPAI mutation, but it has been linked to the introduction of the LPAI virus from wild birds into poultry farms^6^. The more the LPAI viruses circulate and replicate in poultry dense areas, the higher the risk of mutation to HPAI viruses^6^. LPAI viruses circulate in wild bird populations, primarily in waterfowl and migratory water birds belonging to the Anseriform and Charadriiform orders, which are believed to be the major natural LPAI virus reservoirs^7^. In contrast, the main source of HPAI transmission, particularly in Asia and Europe, has been associated with the trade of live poultry, poultry products and smuggling birds^8^. There have been rare reports on the circulation of HPAI in wild birds and the associated prevalence and pathogenicity in those HPAI wild bird cases has been shown to vary widely depending on the infected species^9^.

Historically, the US was affected by several HPAI epidemics; the most important ones occurred in 1924, 1983, 2004 and recently in 2014–2015^10^,^11^. There were no reports of significant human illness resulting from any of these outbreaks^12^. The 1924 H7 outbreak involved East Coast live bird markets^13^,^14^ but was successfully controlled and eradicated at an estimated cost of $^13^ million^15^. The 1983–84 H5N2 outbreak resulted in the destruction of approximately 17 million chickens, turkeys and guinea fowl in the northeastern US to contain and eradicate the disease^16^. In 2004, the USDA confirmed an H5N2 outbreak in chickens in the southern US^17^,^18^. The outbreak was quickly controlled due to close coordination and cooperation between the USDA and state and industry leaders. The recent 2014–2015 HPAI epidemic affected over 21 states, where 242 poultry farms (221 commercial, 21 backyard) and around 100 wild birds were confirmed to be HPAI positive^19^,^20^. The estimated costs to control the outbreak exceed $950 million^19^. Although the main sources of disease introduction and spread of this 2014–2015 epidemic are still under investigation, genetic analysis revealed the presence of different HPAI subtypes, including H5N8, H5N2 and a novel H5N1, which has been attributed as the likely cause of the recent epidemic^19^. This new H5N1 strain is distinct from those found in other parts of the world. USDA investigations suggest that this new H5N1 is the consequence of a re-assortment between Asian H5N8 HPAI strains with North American LPAI viruses and probably occurred during summer arctic migration, where Asian and Alaskan migratory birds intermingle.

The first confirmed case of the U.S. 2014–2015 epidemic occurred on December 2014 at a small-scale backyard operation in Oregon consisting of approximately 130 poultry birds^21^. The first wild bird cases were detected in Washington state in December 2014^22^. Several outbreaks were subsequently reported in wild bird populations in addition to multiple backyard and commercial poultry flocks in the West Coast. In April 2015, cases were observed in the American Midwest in Wisconsin where a State of Emergency was declared as a response to the rapid spread of the AI viruses^23^. The states of Iowa, Minnesota and Wisconsin were most affected representing 90% of the total number of birds infected across the U.S. Overall, over 232 outbreaks occurred during 2014–2015 affecting almost 50 million birds^24^. Preliminary studies show that the recent HPAI outbreaks are linked to the North American Migratory Flyways [NAMF]^25^,^26^. These flyways are migratory paths used by bird species moving between Canada, Latin America or Asia in search of water and food. Interestingly, some AIV strains are found only in specific flyways^27^. Flyways have specific climatic and environmental characteristics that may impact the AI epidemiological cycle in these areas. A better understanding of the factors contributing to the LPAI presence and occurrence of HPAI outbreaks in the different flyway sub-regions as well as the identification of areas at highest risk of AIV transmission, especially at the wild-domestic interface, are needed. This knowledge could be used to develop and implement solutions for improved prevention and rapid control of future HPAI epidemics in the US.

Species Distribution Modeling (SDM) has proven to be effective for identifying the most important predictors contributing to HPAI outbreaks and for determining likely areas of HPAI occurrence in previous studies in Japan and the Middle East^28^,^29^. Moreover, areas combining high poultry density with suitable areas for LPAI may be at higher risk of mutation from LPAI to HPAI. In this study we aim to prove the value of using LPAI suitability maps based on wild birds LPAI surveillance in addition to climatic, environmental and, poultry demographic factors to identify areas at high risk of HPAI outbreak occurrence. A “presence-only” SDM^30^ was used to generate four suitability maps for LPAI considering the four NAMF: Atlantic, Mississippi, Central, and Pacific. These four sub-regions were chosen because (1) they capture the natural migratory bird flyways (i.e., routes the birds follow to migrate between nesting and wintering areas), (2) they correspond to established, non-overlapping, administrative areas^31^, with independent Councils (i.e., representatives from each state and territorial agency and technical committees within each flyway) used to facilitate management of migratory birds and their habitats and, therefore, (3) we believe is the best regionalization for both capturing the distinct eco-epidemiological characteristics of AI and facilitating the decision making and the implementation of potential risk-reduction activities or policies. Results of this study will provide further insights into the epidemiology of AIV in the US and will inform the design of more cost-effective, risk-based surveillance programs specific to each NAMF sub-region for better prevention and control of future HPAI epidemics in the US.

## Results

**Figure 1:**
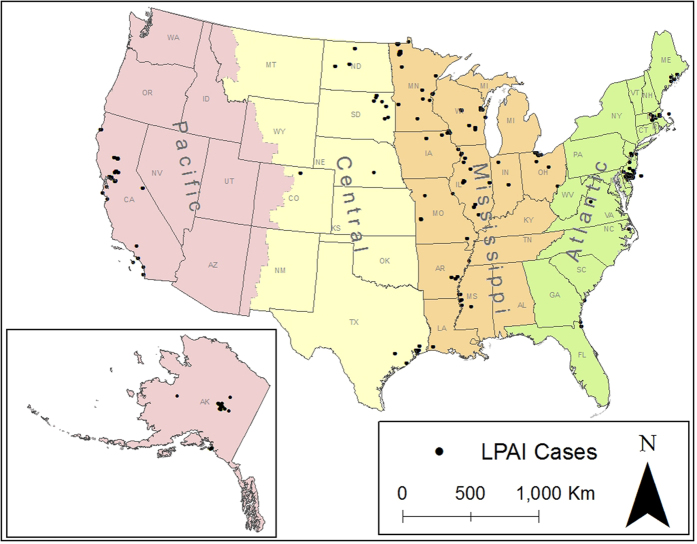
Distribution of the LPAI cases in the United States. The different colors represent the geographic areas covered by the four NAMF according to The U.S. Fish & Wildlife Service 31. The black dots represent the spatial distribution LPAI cases reported by the Influenza Research Database from Jan 2005 to Feb 2015. Maps were created using ArcMap version 10.3 (Environmental Systems Resource Institute, www.esri.com).

The LPAI dataset used in this study consisted of 7,714 positive samples. Those positive samples are spread on the four flyway sub-regions as following: Pacific (37.9%), Central (9.47%), Mississippi (38.96%) and Atlantic (13.67%) (Fig. 1).

**Figure 2:**
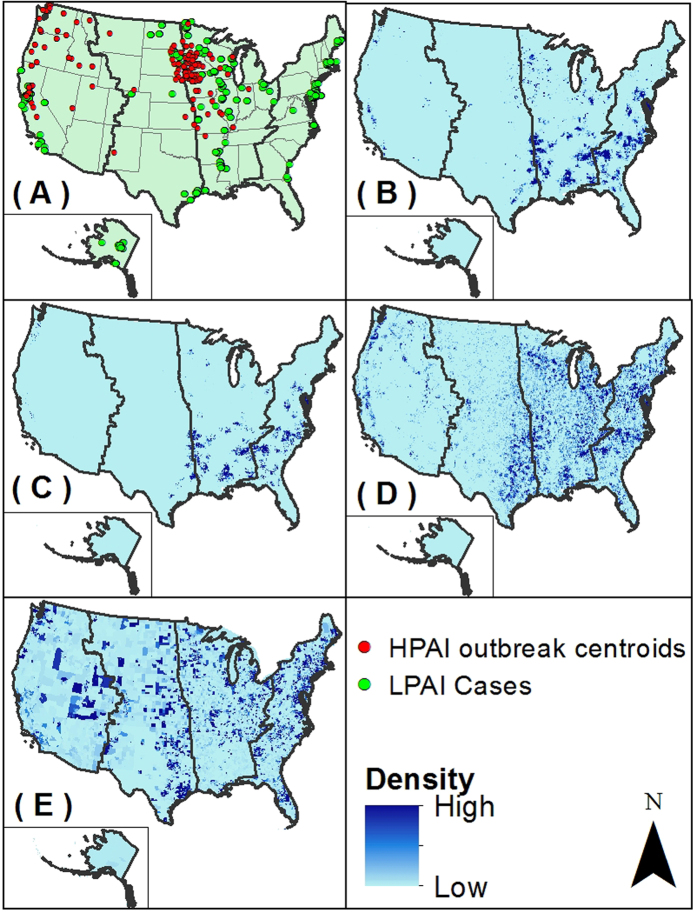
Spatial distribution of the LPAI outbreaks in wild birds from Jan 2005 to Feb 2015 and HPAI 2014–2015 outbreaks in the US. (**A**) Poultry density (**B**), Broiler farm density (**C**), Poultry farm density (**D**), Backyard farm density (**E**). The thick black boundaries represent the limits of each of the four North American Migratory flyways. In map (**A**) green points represent the LPAI samples and the red points represent the HPAI 2014–2015 outbreak centroids. Color gradient of each pixel in map (**B**–**E**) represents density gradient from clear blue shading (low density) to bright blue shading (high density). Maps were created using ArcMap version 10.3 (ESRI, Environmental Systems Resource Institute, www.esri.com).

Spatial distribution of poultry demographics is shown in Fig. 2. Backyard chicken farms were well spread across the country with a higher density in the East Coast region; the Midwest and the Rocky Mountain area have the highest density (Fig. 2E). Poultry farms are abundant in the Atlantic and the Mississippi flyway, Texas and Oklahoma in the Central flyway and the coastal region of the Pacific flyway (Fig. 2D). Poultry density is high in the Atlantic and the Mississippi flyway (Fig. 2B). Broiler farms are located in the South Atlantic in the Atlantic flyway and East South Central in the Mississippi flyway (Fig. 2C).

### Important predictors and high suitable areas for LPAI

**Figure 3:**
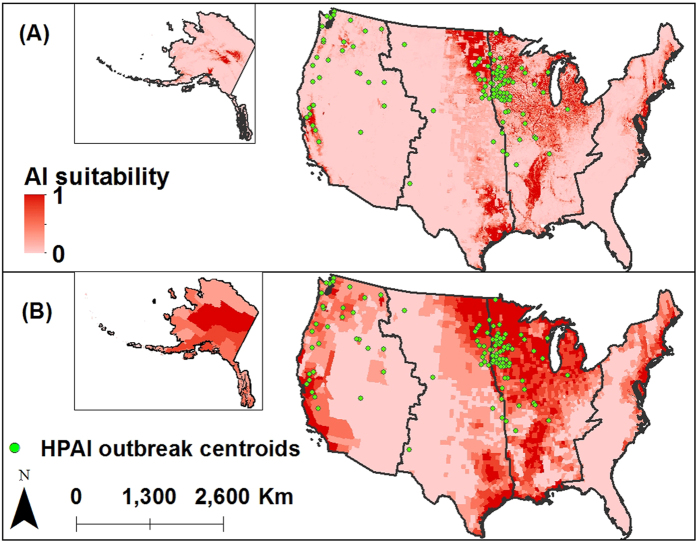
Validation of merged migratory flyways suitability map using the HPAI 2014–2015 outbreak data. The green points represent the centroids of the 2014–2015 HPAI outbreaks. The color gradient of each pixel represents the LPAI presence probability at the county level from clear red shading (low presence probability) to bright red shading (high presence probability). Map was created using both RStudio (RStudio Team, 2015) and ArcMap version 10.3 (ESRI, Environmental Systems Resource Institute, www.esri.com).

Five environmental variables were necessary to determine suitability for LPAI outbreaks in the Pacific flyway with an AUCc of 0.98: altitude, NDVI, mean temperature of the warmest quarter, land cover and backyard chicken farm density (Table 1). Areas of high suitability in the Pacific flyway were the coastal areas in the Pacific North West, the Sacramento Valley and Southern California, the central region in Alaska and the Mid-Atlantic (Fig. 3A). For the Central model, four environmental variables provided a model with an AUCc of 0.94: merged migratory bird abundance, altitude, backyard chicken density and land cover (Table 1). Areas with highest suitability are the eastern part of the coastal plain in Texas and the east areas of South and North Dakota (Fig. 3A). NDVI, land cover, distance to water surfaces and altitude were the variables contributing the most for the Mississippi flyway resulting in a model with an AUCc of 0.88 (Table 1). This model estimated that the North Central States of the Midwest (i.e. Minnesota, Iowa, Michigan, Illinois, Indiana and Ohio) are highly suitable for LPAI (Fig. 3A). For the Atlantic flyway, mean temperature of the warmest quarter, distance to water surfaces, merged migratory bird abundance, backyard chicken farm density and land cover were the most important predictors resulting in a model with an AUCc of 0.96 (Table 1). LPAI suitable areas in the East coast were mostly around the Delaware Bay (Fig. 3A). Overall, very low correlation was observed between variables in each reduced model as shown in the Spearman correlation plots (see Supplementary Figure 1). Response curves also suggested important differences in the ranges in which those variables are important (see Supplementary Figure 2). For example, in the Pacific Flyway, highest relative probability of LPAI occurrence lies between 0 and 1500 meters above sea level, but above 1500 m the relative probability of LPAI occurrence is negligible. Those response curves also reflect a contrast in the range of values that are important when comparing different flyways. For example, type of land cover with the highest relative probability of LPAI presence differs between the four flyways.

### Models’ diagnostics, validation and HPAI prediction ability

Model performance was considered good based on the ROC curves. Jackknives tests on training data, used for model selection, and response curves, used to characterize the areas of highest/lowest relative probability of presence are shown in Supplementary Figure 2 and Supplementary Figure 3. Up to 89% (n = 278/312) of the 2014–2015 HPAI cases were located in counties classified as “highly suitable” in the merged map of the four flyway models.

## Discussion

Results suggest that LPAI surveillance data, together with wild and domestic bird demographics as well as climatic and environmental factors can be used to accurately detect suitable areas for LPAI presence (AUCc from 0.88 to 0.98). Moreover, a total of 89% of the counties reporting HPAI outbreaks during 2014–2015 were in highly suitable areas for LPAI. These results reinforce the hypothesis that the 2014–2015 HPAI outbreaks may have been associated to a LPAI-to-HPAI mutation and transmission at the wild-domestic interface in high bird density areas where high loads of AI viral circulation and replication was occurring. Suitable areas for LPAI presence were concentrated primarily in the North Central region (Minnesota, Iowa, Michigan, Illinois, Indiana and Ohio). Although a previous study in the US reported these states to be at high risk^32^, this study highlights several suitable areas that have not been previously identified (e.g., Coastal Plains of Texas, The Sacramento Valley in California) and that may be under-sampled and under-represented in the current surveillance programs. Moreover, the SDM models provide higher resolution and granularity than previous studies, which were conducted at the county level. Splitting the US into four distinct ecologic and geographic areas following the NAMF is a good method to produce models that capture the particularities of AI epidemiology in those specific regions. Moreover, the characterization of different risk factors in each flyway may facilitate the implementation of specific preventive and risk-mitigation strategies by each of the individual flyway Councils. Bird density rasters generated for this study, particularly backyard poultry farms’ density, provide a novel way to estimate bird populations. Estimates of backyard chicken farms density in California in this study are consistent with surveys conducted by extension specialists to estimate the census of California backyard poultry^33^. The authors believe the backyard bird density raster will benefit future poultry production studies.

Overall, ecological and topographic variables were the main predictors in all of the models presented. More specifically, land cover, altitude, distance to water surface and NDVI were the factors with higher contribution to the LPAI suitability. Consequently, results indicate a higher LPAI suitability in low altitude, cultivated croplands and woody wetlands with a mean temperature of the warmest quarter between 10 and 25 °C. This supports findings of similar studies that considered locations having these conditions as optimal habitats for aggregation of migratory waterfowl and thus at higher risk for AIV transmission between different bird species^28^,^34^. Moreover, humid areas and colder temperatures have been described as favorable for viral persistence in the environment^35^. Land cover was an important contributing factor in all models. Presence of cultivated croplands and woody wetlands seem to offer the most suitable environment for LPAI. In Alaska, areas with Dwarf scrub and Shrub seems to be correlated with LPAI presence^36^. Low altitude was consistently associated with AI outbreaks in three of the four models (Pacific, Central and Mississippi). This reinforces results from previous studies performed in different countries that showed an inverse relationship between altitude and AIV outbreaks^34^,^35^,^37^. Distance to water contributed to the Mississippi and the Atlantic model. Areas close to inland water in the Mississippi region tend to be more suitable for LPAI presence. The Mississippi River basin and associated wetlands have previously been identified as major hotspots for HPAI outbreaks^32^,^38^. These areas contain many shallow bodies of water which have been proven to play an important role in rapid transmission of AIV^39^. The North East of the US, specifically areas around the Delaware Bay and the Delaware River, seems to be highly suitable for LPAI. The Delaware Bay is classified on the Ramsar list of Wetland of International Importance^40^ as it is highly frequented by shorebirds and waterfowls. Those areas were also described as “hotspots” for AI^41^. Areas with moderate NDVI are suitable for LPAI presence in the Mississippi region and areas with low to moderate NDVI seem suitable for LPAI presence in The Pacific region. Low to Moderate NDVI values are often associated with areas where herbaceous plants and grasses are abundant. This type of vegetation represents an optimal food source for some waterfowl. Higher NDVI values are often associated with larger plant and trees^34^. Another predictor associated with the suitability of LPAI was the density of backyard chicken farms in the US^42^, particularly in the Pacific, the Central and Atlantic models (4.7%, 17.1% and 13.1% contribution, respectively). This agrees with previous studies in Asia, the Middle East and Africa that indicate a direct association between high density of backyard chicken and the occurrence of AI outbreaks^43^,^44^,^45^. A better characterization of the spatial distribution and biosecurity of backyard poultry farms will certainly allow refinement of current suitability maps and better identify the specific areas and time periods where AI outbreaks are more likely to occur.

The major challenge faced during this study was obtaining good quality data for the hypothesized important predictors. Namely, the incorporation of more accurate poultry farm location data instead of poultry farm density estimations would improve the ability of the models to identify areas where LPAI transmission from wild birds to poultry or from poultry to wild birds is most likely to occur. Suitability map precision could also be improved with better LPAI and HPAI presence data. Even though certified laboratories collect LPAI data, sampling bias is likely to be present as surveillance efforts are not uniform across all US states (i.e., regions with higher wildlife services stations and poultry production may tend to have more intensive surveillance programs and collect higher numbers of wild bird samples). Furthermore, presence-only models relying on pseudo-absence data were used to correct for the fact that negative samples tend to be under reported by diagnostic laboratories. Positive cases were readily available and are likely to be more complete than negative results. However, adding unbiased negative results to the models could potentially improve the models’ predictions (i.e. using presence-absence MaxEnt). Model validation is restricted by the spatial scale of the HPAI outbreak data available (i.e. data were only available at a county level). Exact HPAI outbreak locations will not only allow a more accurate validation of the LPAI suitability maps but also will be useful for directly generating models using HPAI instead of LPAI data. Similarly, the lack of census data on backyard poultry in the US leads us to generate an estimate based on population census and socio-economic characteristics. Authors believe that using more accurate data on poultry demographics, particularly backyard poultry farms, will increase the predictive ability of our models as well as serve to identify priority areas where outreach and communication strategies should be conducted for backyard producers. Also, the reduced set of wild bird species selected in this study was based on the LPAI prevalence from the Influenza Research Database, which is likely biased and thus may lead to underestimation of the LPAI suitability in some areas. Furthermore, future studies should consider the incorporation of other sources of information on migratory birds’ abundance. The North American Breeding Bird Survey is likely to underrepresent species such as breeding waterfowl that require a different type of sampling. While this dataset might be useful in investigating trends of a proportion of the population over time, it may not be a good indicator of abundance for specific water birds. This could potentially explain why migratory bird density factors were not an important contributing factor as expected by the authors. Future studies should aim to include specific wild bird species density from sources other than the Breeding Bird Survey and to evaluate the relative contribution of each species for each of the migratory flyways models and for different seasons and time periods.

To our knowledge, this is the first study applying SDM to generate high resolution AI suitability maps for the US. These results can be used to better prevent and control future HPAI outbreaks in the US and could be easily extended to other regions in North America. The identification of the main predictors and high suitable areas can be used to implement risk-based and more cost-effective surveillance strategies and to increase awareness of poultry producers located in high suitable zones. The results of this study have been included into a public database within the Disease BioPortal^46^ to allow the visualization of area-specific predictions, the integration of periodic updates and facilitate decision making to minimize the impact of future HPAI epidemics in the US.

## Material and Methods

### Wild bird LPAI presence-only data

Wild bird LPAI positive data was retrieved from the Influenza Research Database (FluDB)^47^. Briefly, FluDB is an influenza database consisting of georeferenced collection coordinates, species names, AI-positive results, viral subtypes and many other collection specifics regarding each bird tested. Samples collected between January 2005 and February 2015 were considered for our study. Samples from domestic species and samples from unknown species or without latitude or longitude coordinates were disregarded (0.2%). A case was defined as a unique animal collected from a specific geographic location that is confirmed to be LPAI positive by necropsy and laboratory testing in one of the NIAID-funded Centers of Excellence for Influenza Research and Surveillance (CEIRS). The CEIRS is a multidisciplinary, collaborative network that aims to control AI nationally and internationally via global surveillance, pathogenesis research and training^48^.

### Environmental data

In total, 14 variables were considered as potential predictors for the models (Table 2). The variables fell into three different categories: (1) climatic, (2) environmental/topographic and (3) domestic and wild bird demographics. All the selected variables were selected based on literature review as they have been described to play an important role for AIV dynamics and distribution in other countries^49^,^50^,^51^.

Spatial data was collected for each of the predictors and 500 m × 500 m cell size rasters were created for each one of them. This resolution was chosen as it offers a good balance between adequate resolution for decision-making and a reasonable processing calculation speed (40 minutes to run one flyway model on average). All the predictor rasters were standardized to have the same map extent and a common projection (NAD_1983_Albers) using ARCGIS 10.3^52^ and RStudio Version 0.98.1102^53^.

Climate data layers were selected from the standard^19^ Bioclim variables from the WorldClim organization^54^,^55^. Bioclim, a set of interpolated climate data, is the result of 50 years of ground-based weather measurements. It contains means of monthly minimum and maximum temperatures and precipitation in a grid format at several different resolutions. As described in previous studies, all the Bioclim variables were included in a preliminary maximum entropy model in order to select the most important predictors as described in previous studies^43^. Based on the result of this preliminary model, the mean diurnal range (Bio2) and mean temperature of warmest quarter (Bio 10) were the sole predictors selected to be included in the final model.

Environmental/topographic variables consisted of altitude, distance to water points, the Normalized Difference Vegetation Index (NDVI) and land cover. Specifically, distance to water consisted of the Euclidean distance to open water (oceans) and to inland water points (rivers, wetlands and lakes). Inland water layers were obtained from US. Geological Survey^56^: the US Lakes and the US Rivers and open water layer was obtained from Natural Earth database^48^. NDVI represent an estimate of vegetation activity measured by the Moderate Resolution Imaging Spectroradiometer (MODIS) aboard NASA’s Terra satellite. NDVI raster incorporated in the models consisted of an average of the vegetation activity estimated over the study period. Land cover at a 5 arc-minutes resolution was obtained from the United States Geological Survey (USGS) database^56^. More details about the different land cover classes and their pixel values in the raster are presented in the Supplementary Table 1. Domestic and wild bird densities were included in the analysis to account for the potential transmission of AIV from wild birds to poultry and *vice versa*. Bird farm density variables included poultry and poultry farm densities in total and by type of production system, the backyard chicken farm density and the migratory bird density. Commercial poultry farm density per km^2^ was generated by the Model of Infectious Disease Agent Study (MIDAS)^57^ using 2002 Census of Agriculture poultry farm counts. This layer contains locations of poultry farms by production system and species (broiler, duck, layer, pullet, turkey). This data was integrated in a Kernel density function with a search radius of 3 km to generate a global density raster for all poultry farms, a raster for the global poultry density and a raster specific to the poultry density in each commercial poultry farming system (broiler, duck, layer, pullet, turkey). Backyard chicken farm density per km^2^ was estimated based on two sources: the percentage of households that owned chickens by race/ethnicity determined using the USDA Agriculture Ownership in Four Inspection U.S. Cities survey conducted during 2010–2012 in four different cities: Denver, Miami, New York city and Los Angeles^58^ and the 2010 human demographic census per census track^59^. Equation (1) used to estimate the number of backyard chicken by census track is:

Equation (1): Backyard chicken estimation by census track


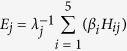


where, *E*_*j*_ is the total estimate of the number of backyard chicken farms per census track per km^2^; *λ*_*j*_ is the area of the census tract in km^2^; *β*_*i*_ is the percentage of race/ethnicity-specific households that owned chickens with values of 0.7 for Asian households (*i* = 1), 0.1 for African-American (*i* = 2), 1.4 for Hispanic/Latino (*i* = 3), 0.7 for White (*i* = 4) and 1.1 for Multiracial (*i* = 5); and *H*_*ij*_ is the number of households by race/ethnicity *i* per census track *j*. Since the census track does not directly provide the number of households by race/ethnicity, only the total number of households and the total population by race/ethnicity were used. *H*_*i*_ was estimated by multiplying the total number of households by the proportion of the population belonging to a particular race/ethnicity for each census track.

Regarding the migratory bird density, only the most relevant wild bird species in terms of AIV risk were considered for this study (i.e., wild bird species with at least 10% LPAI apparent prevalence –number of birds testing positive/total number of birds sampled–) during the study period as observed from the FluDB data). As a result, eight bird species were selected: *Podiceps grisegena* (LPAI prevalence = 0.316); *Bucephala islandica* (LPAI prevalence = 0.182); *Aechmophorus occidentalis* (LPAI prevalence = 0.167); *Bubulcus ibis* (LPAI prevalence = 0.154); *Anas platyrhynchos* (LPAI prevalence = 0.131); *Anas rubripes* (LPAI prevalence = 0.120); *Anas acuta* (LPAI prevalence = 0.102); *Anas discors* (LPAI prevalence = 0.101). Abundance rasters for each of the selected bird species were created from abundance shapefiles extracted from the USGS website^60^. The shapefiles were based on the North American Breeding Bird Survey (1966–2003). Abundance rasters were created for each of the eight migratory birds selected using RStudio (RStudio Team, 2015). Finally, for simplicity, all the individual migratory bird raster’s created were merged into a global unique migratory bird density raster map using the sum of abundance from each individual raster.

### Analysis: Species Distribution Model (SDM)

Presence-only maximum entropy SDM was used to detect areas with high relative probability of LPAI presence and model the top contributing predictors to LPAI suitability. MaxEnt, a maximum entropy approach to presence-only distribution modeling was used for the analysis via the “dismo” package in R software^61^. MaxEnt uses occurrence data (i.e., LPAI confirmed cases) and a set of environmental predictors (climatic, topographic, economic) to determine the geographic distribution of a specific group of individuals in a specific area (here, the LPAI confirmed birds in each of the NAMF). A total of 10,000 background points were randomly chosen for each model. The algorithm behind MaxEnt is described elsewhere^62^,^63^. A default convergence threshold of 0.00001, a regularization of 1 and number of iterations of 500 were chosen for this study. In addition, a logistic model was used so that predictions’ estimates are between 0 and 1 for the spatial suitability per map cell. In this study, four models were generated to predict suitable areas for LPAI in four distinct geographic areas in the US following the NAMF: Atlantic, Central, Mississippi and Pacific flyway^31^. The NAMF correspond to the administrative flyway areas that were established in 1948 based on the migration routes of North American waterfowl and are defined by Councils and Technical Committees that facilitate waterfowl management and conservation across the continent (e.g., hunting regulations, research and habitat management). The goal behind having a model specific to each of these regions is to detect contributing risk factors that may differ or be specific to each sub-region and thus facilitate the implementation of customized interventions for each of them. The administrative flyways of the US are defined as follows: The Atlantic flyway includes the states of Connecticut, Delaware, Florida, Georgia, Maine, Maryland, Massachusetts, New Hampshire, New Jersey, New York, North Carolina, Pennsylvania, Rhode Island, South Carolina, Vermont, Virginia, and West Virginia. The Central flyway which includes the states of Montana, Wyoming, Colorado, New Mexico, Texas, Oklahoma, Kansas, Nebraska, South Dakota, and North Dakota; The Mississippi flyway which includes the states of Alabama, Arkansas, Indiana, Illinois, Iowa, Kentucky, Louisiana, Michigan, Minnesota, Mississippi, Missouri, Ohio, Tennessee, and Wisconsin. The Pacific flyway includes the states of Alaska, Arizona, California, Idaho, Nevada, Oregon, Utah and Washington. These flyways are associated with the major topographic characteristics in North America (Fig. 1). For each of the four flyway models all the 14 variables were included at first in four full-models. Subsequently, variables that contributed to 5% or more for each of the respective “full” models were retained and ran again in so-called “reduced” models. The risk factors selected for each of the “reduced” models were then used to generate the corresponding four suitability maps (Pacific, Central, Mississippi and Atlantic), which were merged together using the Mosaic to New Raster function in ArcMap version 10.3 (ESRI, Environmental Systems Resource Institute) to evaluate the overall AI suitability for US. Spearman’s rank correlation was used to evaluate correlation between predictors and to avoid collinearity problems. Variables with a correlation of 0.5 or higher were considered as highly correlated. In the case that two variables are highly correlated, the variable less related to the outcome is removed.

### Model performance, validation and HPAI prediction ability

Jackknife training gain tests were used to determine which variables have a higher contribution to each model. The jackknife tests were run multiple times in different ways: (i) using all variables, (ii) dropping one variable at a time, and (iii) running the model using only one variable. Variables with the highest training gains or those that reduced the training gain the most when left out of the model are considered to be the most valuable variables to the model. Using k-fold method from the “dismo” package^61^, 80% of the records were used in the construction of the MaxEnt niche models. The remaining 20% of the records were set aside for external validation. The maximum number of iterations for each model was set to 1,000. Both the Area Under the Curve (AUC) of Receiver Operating Characteristics Curve (ROC) and the Calibrated AUC (AUCc) were generated using the “dismo” package^61^ in RStudio^53^ and used to assess the models. The AUCc. previously described^64^ is used to check for spatial sorting bias (i.e., an AUCc close to one shows the absence of spatial sorting bias).

USDA official notifications data of the US HPAI 2014–2015 epidemic were used to evaluate the HPAI outbreak detection rate of the map generated with LPAI data (i.e., the merged flyways). As the HPAI outbreak locations are only available at the county level (county centroids), the first step was to adapt the merged LPAI suitability map considering the maximum suitability present in a county (i.e., assumption of worst case scenario). Counties were then classified as LPAI “high suitable” or “low suitable” based on the median value of the whole dataset. The median for the merged flyways map was 0.3. Therefore, a county was considered as “highly suitable” for LPAI outbreaks if the LPAI suitability probability was above 0.3. In a second step, HPAI outbreak centroids were overlaid over the county level LPAI suitability map. The number of HPAI outbreaks occurring in “high suitable” counties was then calculated.

## Additional Information

**How to cite this article**: Belkhiria, J. *et al.* Application of Species Distribution Modeling for Avian Influenza surveillance in the United States considering the North America Migratory Flyways. *Sci. Rep.* **6**, 33161; doi: 10.1038/srep33161 (2016).

## Supplementary Material

Supplementary Information

## Figures and Tables

**Table 1:** Percent relative contributions of the selected environmental variables to the MaxEnt models.

Variable	Pacific	Central	Mississippi	Atlantic
	% Contribution	% Contribution	% Contribution	% Contribution
Altitude	39.4	17.4	12.5	
Mean Temperature of Warmest Quarter (bio10)	18.7			42.5
Backyard chicken density	4.7	17.1		13.1
Land cover	18.4	10.4	27.2	12.3
Distance to water surfaces			21.5	17.4
NDVI	18.8		38.8	
Merged Migratory birds abundance		55.2		14.6
**AUC**	**0.98**	**0.94**	**0.88**	**0.96**
**AUCc**	**0.98**	**0.90**	**0.90**	**0.97**

**Table 2:** Variables considered in the model.

Variable	Category	Source
Mean Diurnal Range	Climate	http://www.worldclim.org
Mean Temperature of Warmest Quarter	Climate	http://www.worldclim.org
Altitude	Topographic	http://www.worldclim.org
Land cover	Topographic	http://nationalmap.gov
Distance to water point	Topographic	http://nationalmap.gov; http://www.naturalearthdata.com
Normalized Difference Vegetation Index (NDVI)	Topographic	http://neo.sci.gsfc.nasa.gov
Backyard chicken	Poultry density	http://usdasearch.usda.gov; https://www.nhgis.org
Poultry density	Poultry density	http://www.epimodels.org
Poultry farm density	Poultry density	http://www.epimodels.org
Broiler density	Poultry density	http://www.epimodels.org
Ducks density	Poultry density	http://www.epimodels.org
Layers density	Poultry density	http://www.epimodels.org
Pullets density	Poultry density	http://www.epimodels.org
Turkey density	Poultry density	http://www.epimodels.org
Merged Migratory birds density	Wild Bird density	http://www.mbr-pwrc.usgs.gov

## References

[b1] World Health Organization & World Health Organization. Avian Influenza: assessing the pandemic threat.Technical Report. Available at: http://www.who.int/influenza/resources/documents/h5n1_assessing_pandemic_threat. (Accessed: 02 March 2016) (2005).

[b2] Elçi C. The impact of HPAI of the H5N1 strain on economies of affected countries. International Conference on Human and Economic Resources, 102–115 (2006).

[b3] Avian Influenza Virus (H5N1): a Threat to Human Health. Clinical Microbiology Reviews 20, 243 (2007). 10.1128/CMR.00037-06 PMC1865597 17428885

[b4] Alexander D. J. An overview of the epidemiology of avian influenza. Vaccine 25, 5637–5644 (2007). 17126960 10.1016/j.vaccine.2006.10.051

[b5] Avian influenza virus hemagglutinins H2, H4, H8, and H14 support a highly pathogenic phenotype. *Proceedings of the National Academy of Sciences* 109, 2579 (2012). 10.1073/pnas.1109397109 PMC3289369 22308331

[b6] Capua I. & Alexander D. J. Avian influenza infections in birds–a moving target. Influenza and other respiratory viruses 32(4), 275–286 (2007). 10.1111/j.1750-2659.2006.00004.x PMC4634665 19459279

[b7] Olsen B. *et al.* Global patterns of influenza a virus in wild birds. Science 312, 384–8–388 (2006). 10.1126/science.1122438 16627734

[b8] Webster R. G., Bean W. J., Gorman O. T., Chambers T. M. & Kawaoka Y. Evolution and ecology of influenza A viruses. Microbiol Rev 56, 152–79–179 (1992). 10.1128/mr.56.1.152-179.1992 PMC372859 1579108

[b9] Hénaux V. Model-Based Evaluation of Highly and Low Pathogenic Avian Influenza Dynamics in Wild Birds. PLoS ONE 5, e10997 (2010). 20585637 10.1371/journal.pone.0010997 PMC2890401

[b10] Highly pathogenic avian influenza spreads in the USA. Veterinary Record 176, 505 (2015). 25977485 10.1136/vr.h2582

[b11] Clement T., Nezworski J., Scaria J., Nelson E. & Christopher-Hennings J. Complete Genome Sequence of a Highly Pathogenic Avian Influenza Virus (H5N2) Associated with an Outbreak in Commercial Chickens, Iowa, USA, 2015. Genome Announcements 3, e00613–15 (2015). 26067961 10.1128/genomeA.00613-15 PMC4463525

[b12] HarlessA & Usda. United states prepares for highly pathogenic H5N1 avian influenza in wild birds. Archived USDA Fact Sheet No. 0092.06. Available at: http://www.usda.gov/wps/portal/usda/usdamediafb?contentid=2006/03/0092.xml&printable=true (Accessed: 02 March 2016).

[b13] Lupiani B. & Reddy S. M. The history of avian influenza. Comparative immunology, doi: 10.1016/j.cimid.2008.01.004 (2009). 18533261

[b14] Wood J. M., Webster R. G. & Nettles V. F. Host range of A/Chicken/Pennsylvania/83 (H5N2) influenza virus. Avian Diseases 29, 198–207 (1985). 3985875

[b15] Swayne D. E. Diseases of Poultry13th edn. Section II, Ch.6, Table 6.1, page 183 (2013).

[b16] Hinshaw V. S., Nettles V. F., Schorr L. F., Wood J. M. & Webster R. G. Influenza Virus Surveillance in Waterfowl in Pennsylvania after the H5N2 Avian Outbreak. Avian Diseases 30, 207 (1986). 3015104

[b17] Lee C.-W., Swayne D. E., Linares J. A., Senne D. A. & Suarez D. L. H5N2 Avian Influenza Outbreak in Texas in 2004: the First Highly Pathogenic Strain in the United States in 20 Years? Journal of Virology 79, 11412–11421 (2005). 16103192 10.1128/JVI.79.17.11412-11421.2005 PMC1193578

[b18] Pelzel A. M., McCluskey B. J. & Scott A. E. Review of the highly pathogenic avian influenza outbreak in Texas, 2004. J Am Vet Med Assoc 228, 1869–75 (2006). 16784375 10.2460/javma.228.12.1869

[b19] Services, V. & USDA. 2016 HPAI Preparedness and Response Plan. Technical report. (2016) Available at: https://www.aphis.usda.gov/animal_health/downloads/animal_diseases/ai/hpai-preparedness-and-response-plan-2015.pdf. (Accessed: 02 March 2016).

[b20] United States Department of Agriculture. Wild Bird Highly Pathogenic Avian Influenza Cases In The United States. Technical report. (2015) Available at: https://www.aphis.usda.gov/animal_health/downloads/animal_diseases/ai/hpai-preparedness-and-response-plan-2015.pdf. (Accessed: 02 March 2016).

[b21] Pokarney B. News release: Oregon activates avian influenza response plan. Report. (2014). Technical report. (2014) Available at: http://odanews.wpengine.com/news-release-oregon-activates-avian-influenza-response-plan/. (Accessed: 02 March 2016).

[b22] Aphis & USDA. Wild Birds Highly Pathogenic Avian Influenza cases in the United States. Technical Report (2015). Available at : https://www.aphis.usda.gov/wildlife_damage/downloads/WILD%20BIRD%20POSITIVE%20HIGHLY%20PATHOGENIC%20AVIAN%20INFLUENZA%20CASES%20IN%20THE%20UNITED%20STATES.pdf. (Accessed: 02 March 2016).

[b23] Mace, M., USDA Situation Report. Technical report. (2015) Available at: https://jeffersoncountyapps.jeffersoncountywi.gov/jc/public/customPrograms/weekly_meeting.php?file=/UserFiles/County%20Board/files/Handout/2015/06152015/LWCC%20Handout.pdf (Accessed: 02 March 2016).

[b24] United States Department of Agriculture. USDA-Confirmed Avian Influenza detections. Update on Avian Influenza Findings Poultry Findings Confirmed by USDA’s National Veterinary Services Laboratories. (2016) Available at: https://www.aphis.usda.gov/aphis/ourfocus/animalhealth/animal-disease-information/avian-influenza-disease/sa_detections_by_states/ai-2016-map (Accessed: 02 March 2016).

[b25] United States Department of Agriculture. Surveillance Plan for Highly Pathogenic Avian Influenza in Waterfowl in the United States. national flyway council report (2015). Available at: https://www.aphis.usda.gov/animal_health/downloads/animal_diseases/ai/2015-hpai-surveillance-plan.pdf (Accessed: 02 March 2016).

[b26] Greene, J. L., Update on the Highly-Pathogenic Avian Influenza Outbreak of 2014-2015. Congressional service report (2015). Available at: (https://www.fas.org/sgp/crs/misc/R44114.pdf (Accessed: 02 March 2016).

[b27] Spackman E. *et al.* Phylogenetic analyses of type A influenza genes in natural reservoir species in North America reveals genetic variation. Virus Res 114, 89–100 (2005). 16039745 10.1016/j.virusres.2005.05.013

[b28] Moriguchi S., Onuma M. & Goka K. Potential risk map for avian influenza A virus invading Japan. Diversity and Distributions 19, 78–85 (2013).

[b29] Adhikari D. & Barik K. Modelling the ecology and distribution of highly pathogenic avian influenza (H5N1) in the Indian subcontinent. Current Science 97, 1, 10 (2009).

[b30] Phillips S. J., Anderson R. P. & Schapire R. E. Maximum entropy modeling of species geographic distributions. Ecological modelling 190, 231–259 (2006).

[b31] U.S. Fish and Wildlife Service. Administrative Flyways. Available at: https://www.fws.gov/birds/management/flyways.php. (Accessed: 02 March 2016).

[b32] Fuller T. L. *et al.* Mapping the risk of avian influenza in wild birds in the US. BMC Infect Dis 10, 187 (2010). 20573228 10.1186/1471-2334-10-187 PMC2912310

[b33] University of California Cooperative Extension. California Backyard Poultry Census. Available at: http://ucanr.edu/sites/poultry/California_Poultry_Census/ (Accessed: 02 March 2016).

[b34] Si Y., Wang T., Skidmore A. K., de Boer W. F. & Li L. Environmental factors influencing the spread of the highly pathogenic avian influenza H5N1 virus in wild birds in Europe. Ecol Soc 15, 26 (2010).

[b35] Brown J. D., Goekjian G., Poulson R. & Valeika S. Avian influenza virus in water: infectivity is dependent on pH, salinity and temperature. Veterinary Microbiology, 136, 20–26 (2009). 19081209 10.1016/j.vetmic.2008.10.027

[b36] Herrick K. A., Huettmann F. & Lindgren M. A. A global model of avian influenza prediction in wild birds: the importance of northern regions. Vet Res 44, 42 (2013). 23763792 10.1186/1297-9716-44-42 PMC3687566

[b37] Loth L., Gilbert M., Osmani M. G. & Kalam A. M. Risk factors and clusters of highly pathogenic avian influenza H5N1 outbreaks in Bangladesh. Preventive veterinary Medicine 96, 104–113 (2010). 20554337 10.1016/j.prevetmed.2010.05.013 PMC4874198

[b38] Khaliq Z., Leijon M., Belák S. & Komorowski J. A complete map of potential pathogenicity markers of avian influenza virus subtype H5 predicted from 11 expressed proteins. BMC Microbiology 15, 128 (2015). 26112351 10.1186/s12866-015-0465-x PMC4482282

[b39] Hanson B. A., Luttrell M. P., Goekjian V. H. & Niles L. Is the occurrence of avian influenza virus in Charadriiformes species and location dependent? Journal of Wildlife Diseases 44, 351–361 (2008). 18436667 10.7589/0090-3558-44.2.351

[b40] Wetland International, Ramsar site database. Ramsar Sites Criteria. URL http://www.ramsar.org/ (Accessed: 02 March 2016).

[b41] Stallknecht D. E. *et al.* Detection of avian influenza viruses from shorebirds: Evaluation of surveillance and testing approaches. Journal of Wildlife Diseases 48, 382 (2012). 22493113 10.7589/0090-3558-48.2.382 PMC3584701

[b42] Beam A., Garber L., Sakugawa J. & Kopral C. Salmonella awareness and related management practices in US urban backyard chicken flocks. Prev Vet Med 110, 481–488 (2013). 23290129 10.1016/j.prevetmed.2012.12.004

[b43] AlKhamis M. A., Hijmans R. J. & Al A. The use of spatial and spatio-temporal modeling for surveillance of H5N1 highly pathogenic avian influenza in poultry in the Middle East. Avian Diseases 60, 146–155 (2016). 27309050 10.1637/11106-042115-Reg

[b44] Paul M., Wongnarkpet S., Gasqui P. & Poolkhet C. Risk factors for highly pathogenic avian influenza (HPAI) H5N1 infection in backyard chicken farms, Thailand. Acta tropica 118, 209–216 (2011). 21459074 10.1016/j.actatropica.2011.03.009

[b45] Chotpitayasunondh T. & Ungchusak K. Human disease from influenza A (H5N1), Thailand, 2004. Emerg Infect Dis 11, 201–209 (2005). 15752436 10.3201/eid1102.041061 PMC3320461

[b46] Center for Animal Disease Modeling and Surveillance., Disase BioPortal. Public Database. (2016) Available at: http://bioportal.ucdavis.edu/about (Accessed: 02 March 2016).

[b47] Influenza Research Database. Public Database. (2016) Available at: http://www.fludb.org/brc/home.spg?decorator=influenza (Accessed: 02 March 2016).

[b48] Natural Earth raster (2016). Available at: http://www.naturalearthdata.com. (Accessed: 02 March 2016).

[b49] Fang L.-Q. *et al.* Environmental Factors Contributing to the Spread of H5N1 Avian Influenza in Mainland China. PLoS ONE 3, e2268 (2008). 18509468 10.1371/journal.pone.0002268 PMC2386237

[b50] Maftei D., Apostu C. & Suru A. Environmental and anthropogenic risk factors for highly pathogenic avian influenza subtype H5N1 outbreaks in Romania, 2005–2006. Veterinary Research Communications 32, 627 (2008). 18528778 10.1007/s11259-008-9064-8

[b51] Mapping H. 5. N. 1. highly pathogenic avian influenza risk in Southeast Asia. Proceedings of the National Academy of Sciences 105, 4769 (2008). 10.1073/pnas.0710581105 PMC2290786 18362346

[b52] Esri Inc. ArcGIS Desktop: Release 10.3 Redlands, CA: Environmental Systems Research Institute. Available at: http://www.esri.com/ (Accessed: 02 March 2016).

[b53] WorldClim. Global Climate Data (2016). Available at: http://worldclim.com/ (Accessed: 02 March 2016).

[b54] Hijmans R. J., Cameron S. E. & Parra J. L. Very high resolution interpolated climate surfaces for global land areas. International journal of Climatology 25, 1965–1978 (2005).

[b55] United States Geological survey. The National Map Small Scale. USGS database. (2016) Available at: http://nationalmap.gov/small_scale/atlasftp.html (Accessed: 02 March 2016).

[b56] Models of Infectious Disease Agent Study. Midas Synthetic Ecosystems database. (2008) Available at: http://www.epimodels.org/drupal/?q=node/32 (Accessed: 02 March 2016).

[b57] United States Department of Agriculture. USDA-Agriculture Ownership in Four Inspection U.S. Cities. Technical report. (April 2013). Available at : https://www.aphis.usda.gov/animal_health/nahms/poultry/downloads/poultry10/Poultry10_dr_Urban_Chicken_Four.pdf (Accessed: 02 March 2016).

[b58] National Historical Geographic Information System. NHGIS US population demographic database. (2016) Available at: https://www.nhgis.org/ (Accessed: 02 March 2016).

[b59] United States Geological survey. Geographic Information Products from the North American Breeding Bird Survey Version 2004.1.(2006) Available at : http://www.mbr-pwrc.usgs.gov/bbs/geographic_information/geographic_information_products_.htm (Accessed: 02March 2016).

[b60] Hijmans R. J., Phillips S., Leathwick J. & Elith J. Dismo: Species Distribution Modeling. R package Version 1.0-15. (2016) Available at: https://cran.r-project.org/web/packages/dismo/vignettes/sdm.pdf (Accessed: 02 March 2016).

[b61] Phillips S. J., Dudík M. & Schapire R. E. A maximum entropy approach to species distribution modeling. Proceedings of the Twenty-First international conference on Machine Learning 655–662 (2004).

[b62] Olden J. D., Lawler J. J. & Poff N. L. Machine learning methods without tears: a primer for ecologists. Q Rev Biol 83, 171–193 (2008). 18605534 10.1086/587826

[b63] R Foundation for Statistical Computing. R Core Team (2013) Vienna, Austria. Available at: http://www.R-project.org. (Accessed: 02 March 2016).

[b64] Hijmans R. J. Cross-validation of species distribution models: removing spatial sorting bias and calibration with a null model. Ecology 93, 679–688 (2012). 22624221 10.1890/11-0826.1

