# Supplementary material for: Application of Species Distribution Modeling for Avian Influenza surveillance in the United States considering the North America Migratory Flyways
Source: Sci Rep. 2016 Sep 14;6:33161. doi: 10.1038/srep33161 (PMC5021976; doi:10.1038/srep33161)
Supplement: Supplementary Information [file srep33161-s1.pdf]

Application of Species Distribution Modeling for Avian Influenza surveillance in the United States considering the North America Migratory Flyways.

Jaber Belkhiria[1*], Moh A. Alkhamis[2,3], Beatriz Martínez-López[1]

[1]Center for Animal Disease Modeling and Surveillance, Department of Medicine & Epidemiology, School of Veterinary Medicine, University of California Davis, California, United States of America

[2]Environmental & Life Sciences Research Center, Kuwait Institute for Scientific Research, Kuwait

[3]Veterinary Population Medicine Department, Veterinary Medical Center, University of Minnesota, St. Paul, Minnesota, United States of America

*Corresponding author: E-mail: Jabelkhiria@ucdavis.edu

Supplementary Table 1. Landcover classes and their pixel values in the raster used in the models. Source: http://nationalmap.gov.

Supplementary Figure 1. Spearman Correlation plots for the four migratory flyway "reduced" models (Pacific, Central, Mississippi, and Atlantic). Plots were created using RStudio (RStudio Team, 2015).

Supplementary Figure 2. Jackknife of Regularized Training Gain of the variables included in each of the four migratory flyways "reduced" models (Pacific, Central, Mississippi, and Atlantic). The red bar represents the overall training gain with all the included variables. The blue bar represents the training gain when using each variable in isolation. The clear blue bar is the training gain when the variable is excluded from the model. Plots were created using RStudio (RStudio Team, 2015).

Supplementary Figure 3. Response curve for each of the four migratory flyway "reduced" models (Pacific, Central, Mississippi, and Atlantic). Plots were created using RStudio (RStudio Team, 2015).

Supplementary Table.1

Pixel Value	Land cover classes	Pixel Value	Land cover classes
0	Ocean water	51	Dwarf scrub (Alaska only)
11	Open water	52	Shrub/ scrub
12	Perennial ice/ snow	71	Grassland/ herbaceous
21	Developed, open space	72	Sedge/ herbaceous (Alaska only)
22	Developed, low intensity	74	Moss (Alaska only)
23	Perennial ice/ snow	81	Pasture hay
24	Developed, high intensity	82	Cultivated crops
31	Barren land	90	Woody wetlands
41	Deciduous forest	95	Emergent herbaceous wetlands
42	Evergreen forest	101, 102, 103	Non-Us. Land
43	Mixed forest		

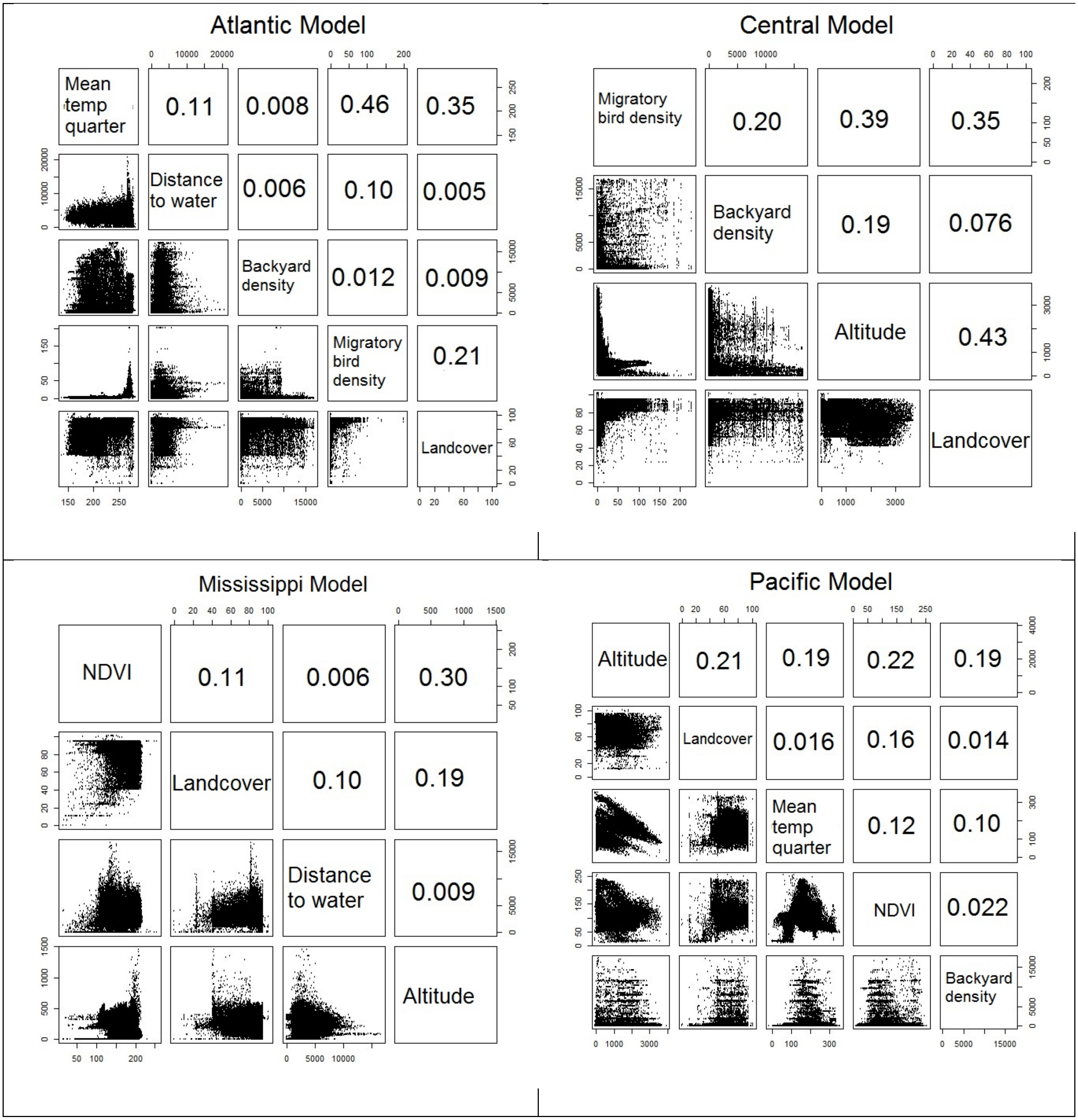

Supplementary Figure 1

Atlantic Flyway

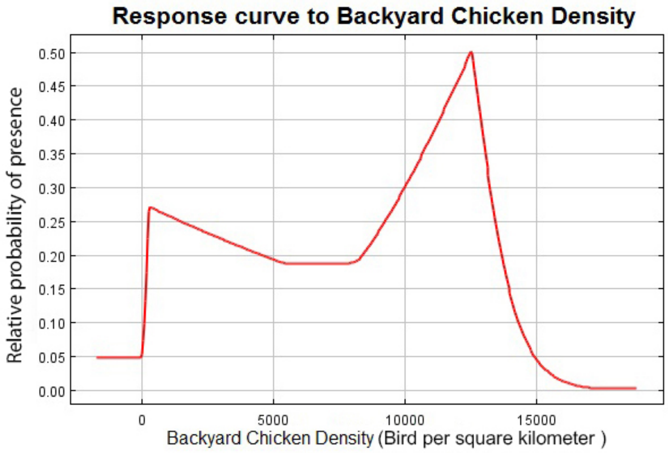

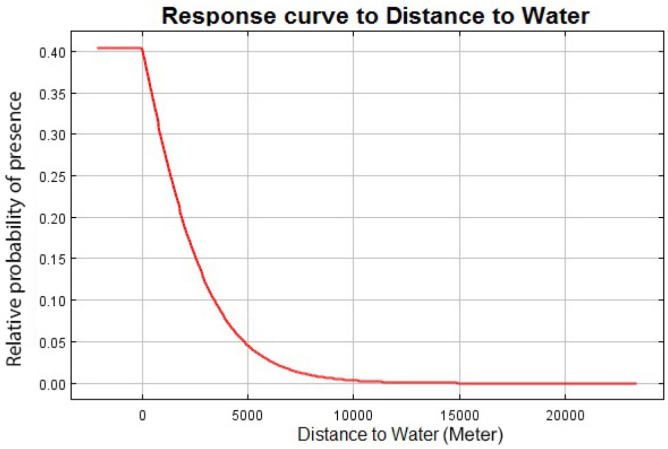

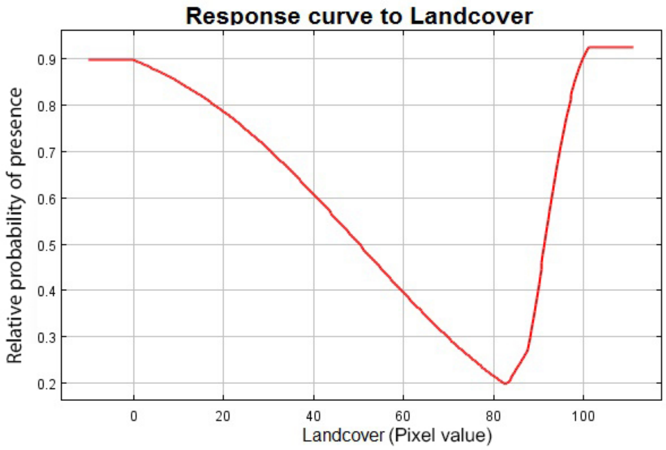

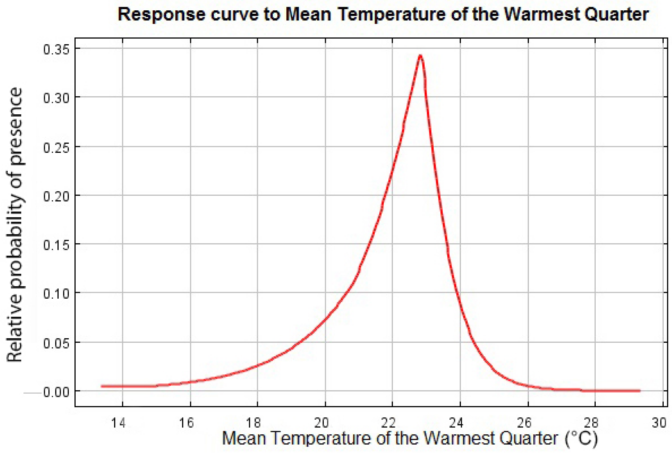

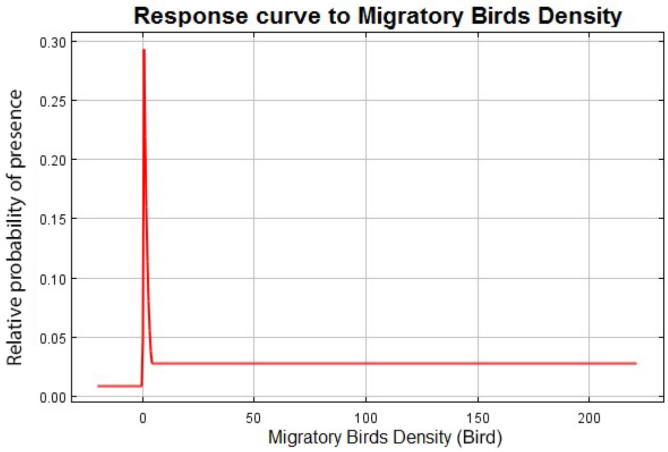

Central Flyways

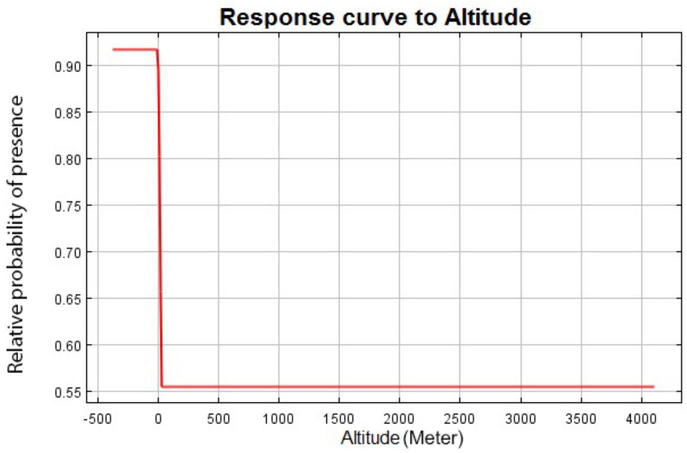

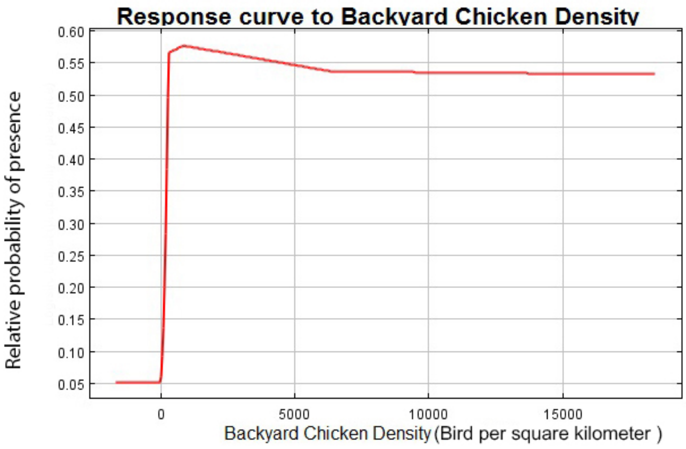

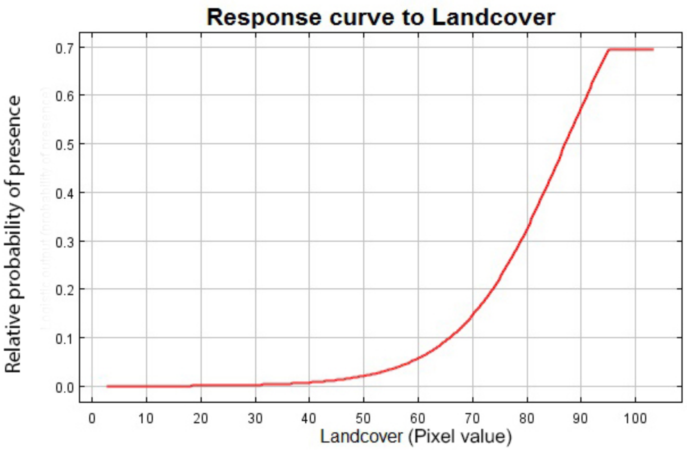

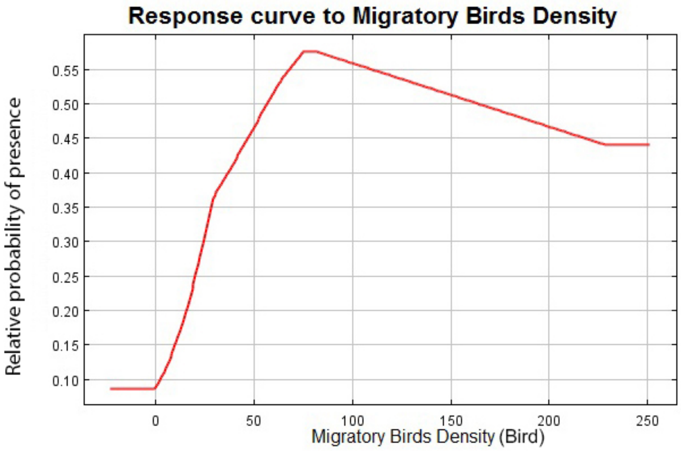

Mississippi Flyway

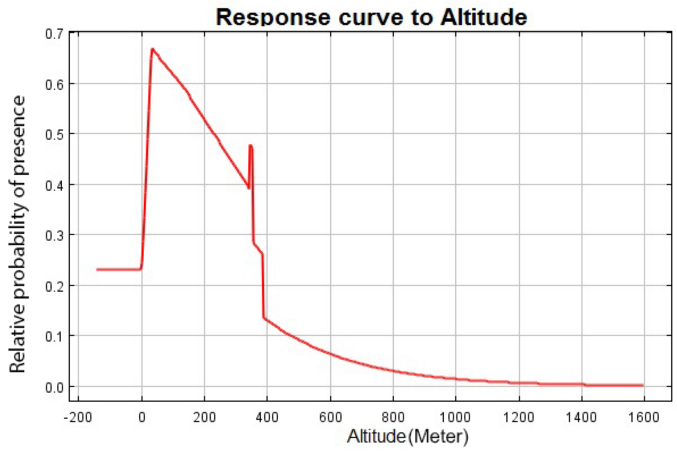

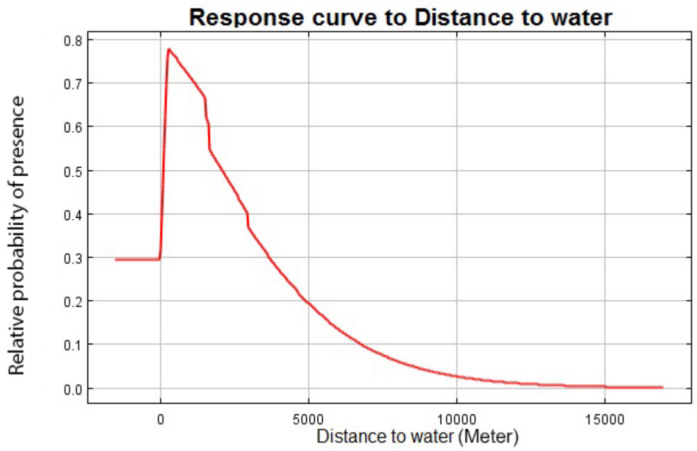

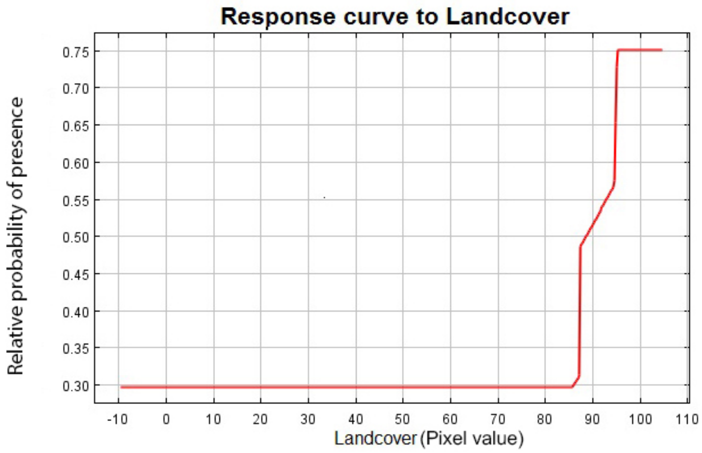

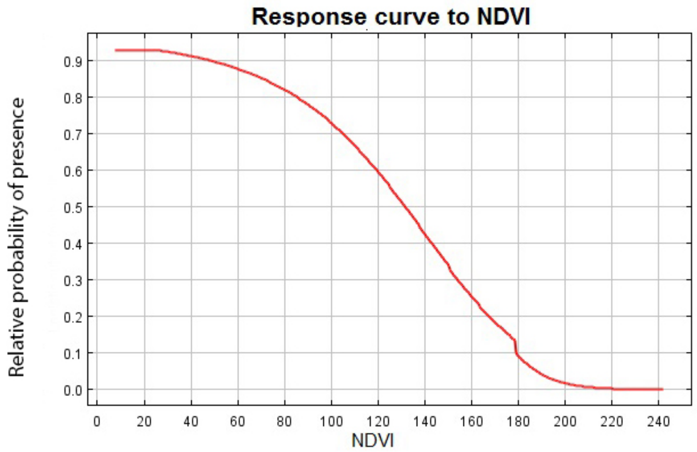

Pacific Flyway

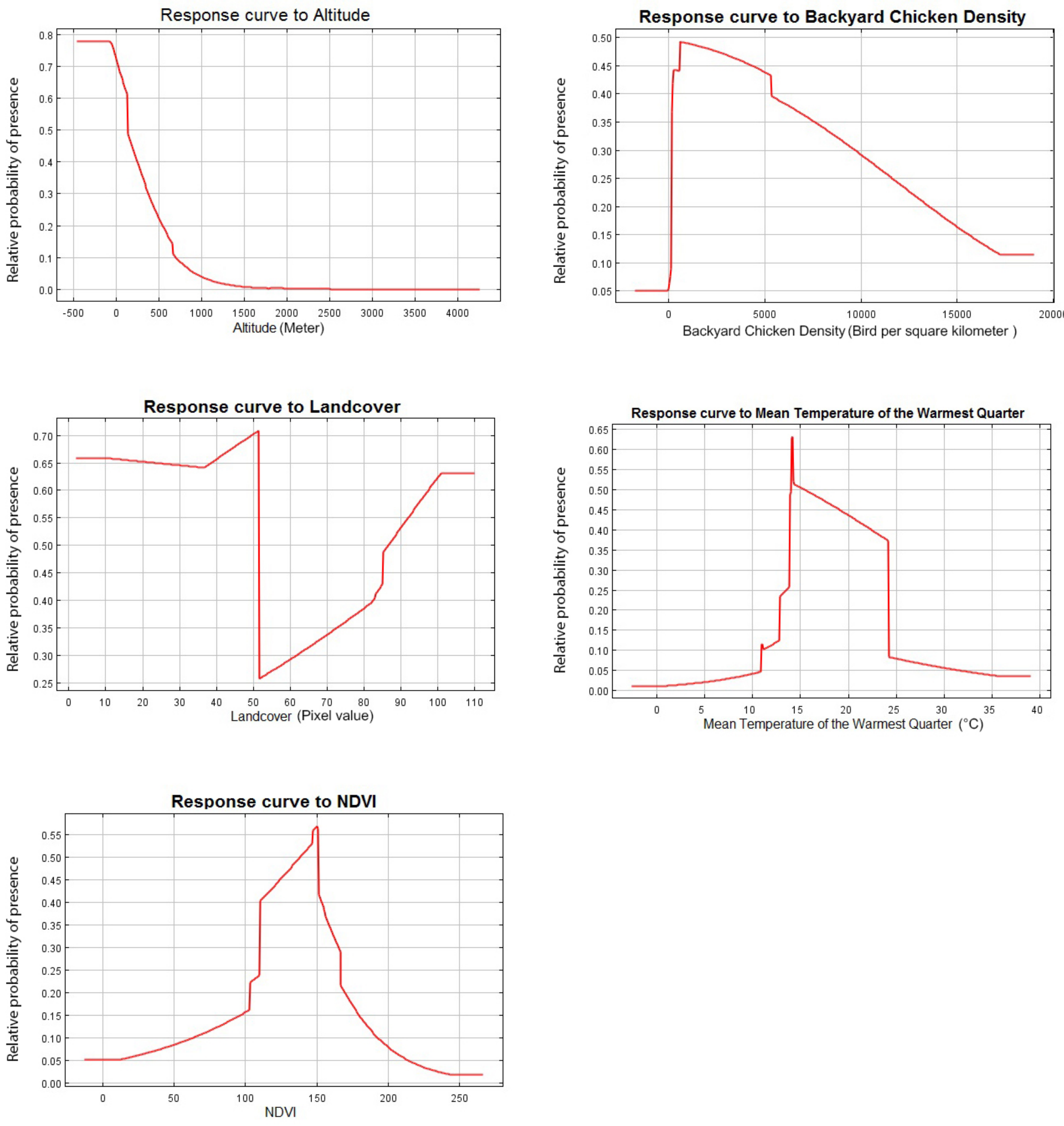

Supplementary Figure 2.

Atlantic Model

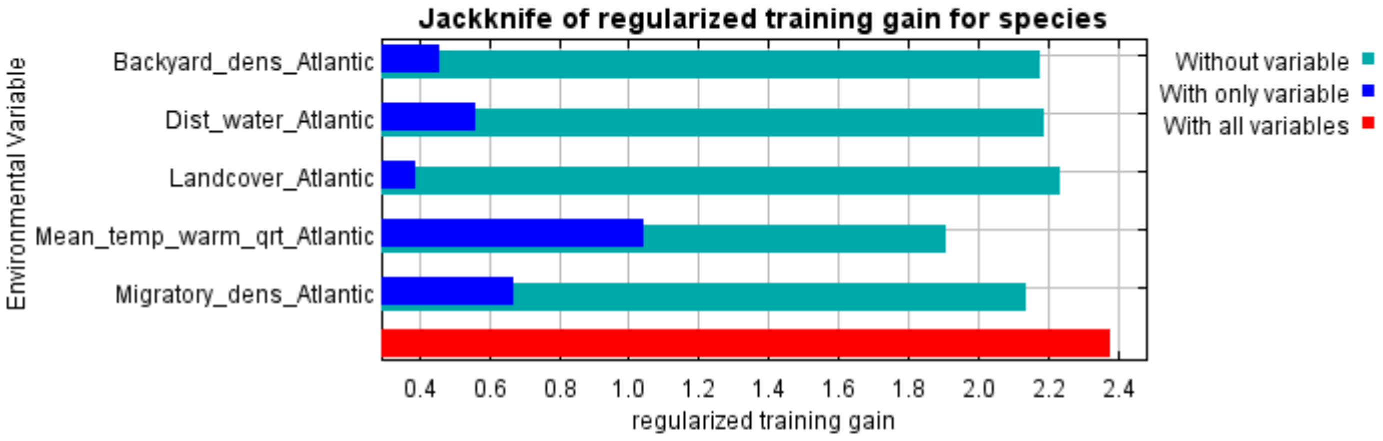

Central Model

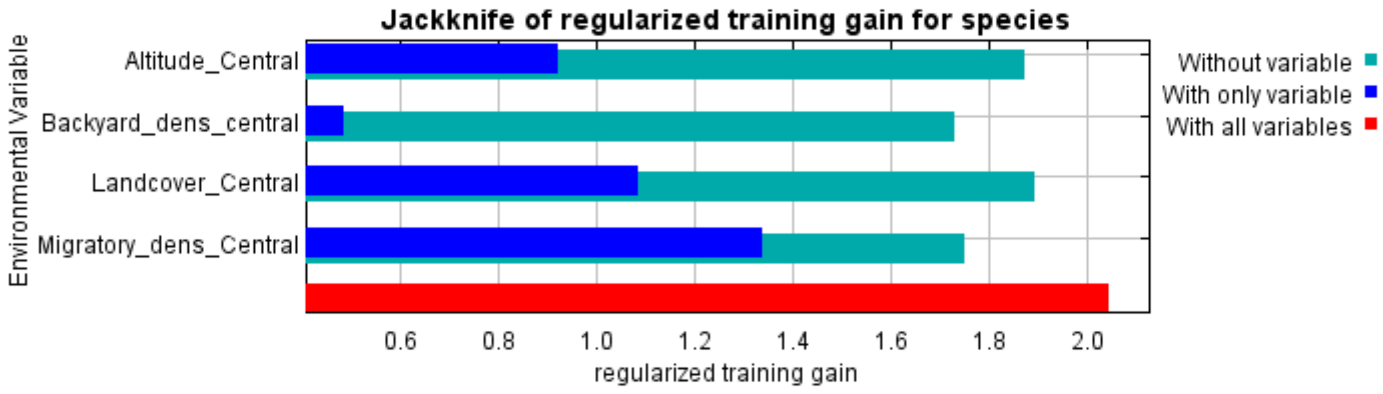

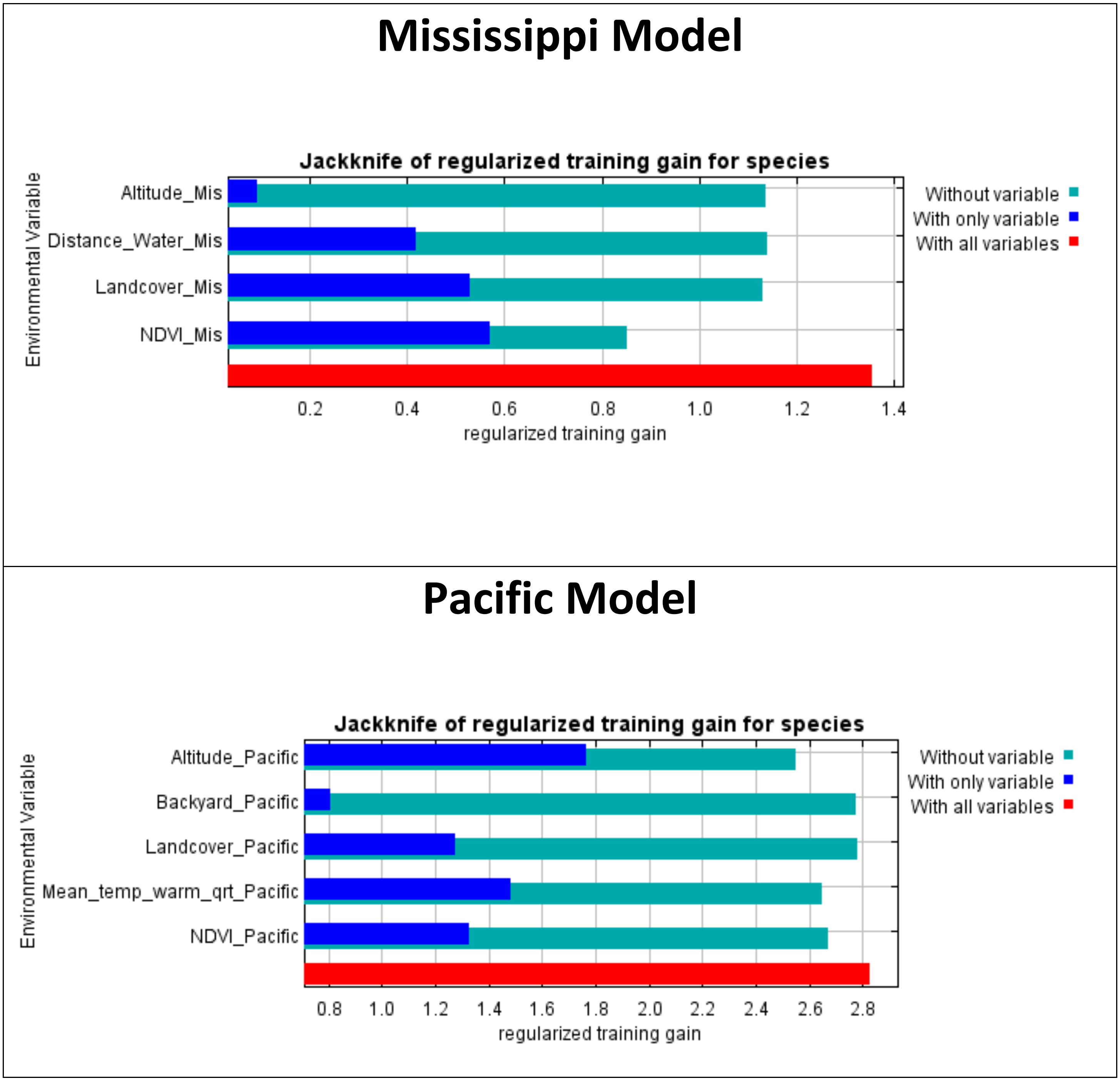

Supplementary Figure 3.
